# metaTraits: a large-scale integration of microbial phenotypic trait information

**DOI:** 10.1093/nar/gkaf1241

**Published:** 2025-11-26

**Authors:** Daniel Podlesny, Chan Yeong Kim, Shahriyar Mahdi Robbani, Christian Schudoma, Anthony Fullam, Lorenz C Reimer, Julia Koblitz, Isabel Schober, Anandhi Iyappan, Thea Van Rossum, Jonas Schiller, Anastasia Grekova, Michael Kuhn, Peer Bork

**Affiliations:** European Molecular Biology Laboratory, Molecular Systems Biology Unit, 69117 Heidelberg, Germany; European Molecular Biology Laboratory, Molecular Systems Biology Unit, 69117 Heidelberg, Germany; European Molecular Biology Laboratory, Molecular Systems Biology Unit, 69117 Heidelberg, Germany; European Molecular Biology Laboratory, Molecular Systems Biology Unit, 69117 Heidelberg, Germany; European Molecular Biology Laboratory, Molecular Systems Biology Unit, 69117 Heidelberg, Germany; Leibniz Institute DSMZ, 38124 Braunschweig, Germany; Leibniz Institute DSMZ, 38124 Braunschweig, Germany; Leibniz Institute DSMZ, 38124 Braunschweig, Germany; European Molecular Biology Laboratory, Molecular Systems Biology Unit, 69117 Heidelberg, Germany; European Molecular Biology Laboratory, Molecular Systems Biology Unit, 69117 Heidelberg, Germany; European Molecular Biology Laboratory, Molecular Systems Biology Unit, 69117 Heidelberg, Germany; European Molecular Biology Laboratory, Molecular Systems Biology Unit, 69117 Heidelberg, Germany; European Molecular Biology Laboratory, Molecular Systems Biology Unit, 69117 Heidelberg, Germany; European Molecular Biology Laboratory, Molecular Systems Biology Unit, 69117 Heidelberg, Germany; Department of Bioinformatics, Biocenter, University of Würzburg, 97074 Würzburg, Germany

## Abstract

Microbes differ greatly in their organismal structure, physiology, and environmental adaptation, yet information about these phenotypic traits is dispersed across multiple databases and is largely unavailable for taxa that remain uncultured. Here, we present metaTraits, a unified and accessible trait resource that integrates culture-derived trait information from Bac*Dive*, BV-BRC, JGI IMG, and GOLD with genome-based predictions for medium and high-quality isolate and metagenome-assembled genomes (MAGs) from proGenomes and SPIRE. metaTraits covers over 2.2 million genomes and >140 harmonized traits mapped to standardized ontologies, spanning cell morphology (e.g. shape, size, and Gram staining), physiology (e.g. motility and sporulation), metabolic and enzymatic activities, environmental preferences (e.g. temperature, salinity, and oxygen tolerance), and lifestyle categories. All records are linked to the original evidence, and species are cross-linked to NCBI and GTDB taxonomies. The interactive metaTraits website provides search and visualization tools, taxonomy-level summaries, and two workflows for annotating user-submitted genomes or community profiles. metaTraits substantially advances accessibility and interoperability of microbial trait data, enabling comprehensive trait-based analyses of microbiomes across diverse environments. metaTraits is accessible via https://metatraits.embl.de.

## Graphical Abstract

**Graphical Abstract:**
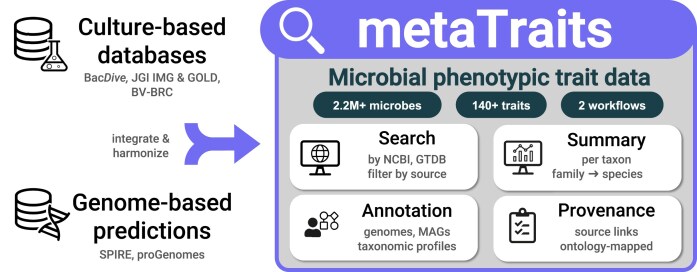

## Introduction

Knowledge of microbial phenotypic traits provides essential insights into microbial functions, ecology, and interactions within both environmental and human-associated ecosystems. Trait information has been used to inform ecological modeling [1, 2], to investigate the codiversification of humans and their gut microbes [3], to characterize diverse global microbial habitats [4], to study functional microbiome shifts in disease contexts [5, 6], and to reveal links between traits and biogeographical and social patterns in microbial strain sharing networks [7, 8], among many other applications. Despite their broad utility and recognized importance, microbial phenotypic trait data remain fragmented across several culture-based repositories, limiting comprehensive and large-scale biological analysis.

Among the most widely used trait databases, Bac*Dive* [9] provides detailed trait data for ~100 000 strains (i.e. records describing individual cultivated microbial entities, with or without genome sequences) from 21 000 species, while resources such as BV-BRC (formerly PATRIC [10]), JGI’s IMG [11], and GOLD [12] also capture trait metadata as part of their genome and sample submissions. However, these databases vary in their data models and curation standards, and trait records are not harmonized across sources, making integration into comparative genomics and microbiome research challenging. As a result, most published analyses rely on a single source and are often limited to well-studied, cultivated isolates.

Several efforts have aimed to unify microbial trait data. Madin *et al.* [13] standardized 26 data sources into a single trait resource for roughly 170 000 microbial records, while BactoTraits [14] mined Bac*Dive* and other datasets to assemble 19 core traits for nearly 20 000 microbes. Earlier projects, such as the Microbe Directory [15], pioneered a community curation approach to trait annotation. Unfortunately, these resources have either not been updated for years, focus exclusively on isolates, cover a limited set of traits, or lack interactive platforms for data exploration and annotation.

Crucially, the vast majority of prokaryotic diversity remains uncultured. Recent estimates suggest that 62% of recognized microbial phyla and 73% of species are represented only by metagenome-assembled genomes (MAGs) [16]. Existing isolate-focused databases thus overlook much of the microbial world, leading to large gaps in trait coverage. Computational methods such as Bac*Dive*-AI [17], GenomeSPOT [18], MICROPHERRET [19], and Traitar [20] have emerged to enable the prediction of microbial traits from genome sequences, potentially extending trait annotation to hundreds of traits (albeit with different accuracy) across millions of publicly available genomes. Yet, no resource systematically integrates both curated culture-derived traits and genome-based predictions across large-scale genome catalogs.

Here, we present metaTraits, a unified and accessible microbial trait resource that harmonizes trait data from major culture-based databases and systematically extends trait annotation to both isolate genomes and MAGs using genome-based prediction tools. metaTraits integrates phenotypic trait data for 2.2 million genomes from over 100 000 SPIRE species-level clusters (≥95% average nucleotide identity), encompassing >140 harmonized traits relevant to microbial morphology, metabolism, lifestyle, and ecology, among others. All data are mapped to standardized ontologies, cross-linked to external resources, and made available in alignment with FAIR principles [21]. By bridging the gap between culture-based and genome-based data, metaTraits enables trait-based microbiome analyses at an unprecedented scale and scope. The resource is accessible via an interactive website that supports user-friendly trait exploration and annotation workflows. metaTraits is openly available at https://metatraits.embl.de.

## Database construction

### Integrating phenotypic trait data from public databases

metaTraits integrates microbial phenotypic trait information from major public databases, including Bac*Dive*, JGI IMG and GOLD, and BV-BRC. The integrated data encompass microbial physiology (e.g. motility and sporulation), environmental preferences (e.g. oxygen requirements, pH, salinity, and temperature), morphology, metabolic and enzymatic activities, and more. Source data were collected via APIs or downloaded as tabular metadata files. To ensure consistency, trait data were manually recoded and converted to common data types and units, with harmonization of trait and category naming, correction of spelling and number formatting errors, and removal of significant outliers. For the isolate-centric databases, metaTraits captures 1 182 280 trait observations across 17 159 species (GTDB; Table 1).

**Table 1.** Overview of microbial phenotypic traits and taxonomy covered by metaTraits

Dataset	Trait groups	Observations/Predictions	Records/Strains	NCBI species	GTDB species
***All***	144	207 340 808	2 238 622	54 654	65 349
***Culture-based records***	58	1 182 280	229 088	25 204	17 159
Bac*Dive*	55	859 472	58 192	14 565	9 468
BV-BRC	11	84 411	18 823	5 153	4 770
JGI IMG/GOLD	20	238 397	152 073	19 247	13 748
***Genome-based predictions***	130	206 158 528	2 009 534	48 029	64 126
proGenomes3	130	68 697 060	906 855	43 305	44 884
SPIREv1	130	137 461 468	1 102 679	14 023	35 416
Genome statistics	6				
Bac*Dive*-AI	9				
GenomeSPOT	10				
MICROPHERRET	52				
Traitar	62				

### Genome-based prediction of phenotypic traits

To address the substantial gaps resulting from uncultured taxa, metaTraits systematically incorporates genome-based phenotypic trait predictions using state-of-the-art computational tools, including Bac*Dive*-AI [17], GenomeSPOT [18], MICROPHERRET [19], and Traitar [20]. These predictions enable extrapolation of phenotypic traits to novel taxa based solely on genomic data, vastly expanding trait coverage beyond what is possible from cultured species alone. As these tools have been trained on different sets of genomes and phenotypes, their predictions inevitably vary in scope and accuracy. To reflect this, metaTraits emphasizes transparency by retaining provenance information for every trait and by summarizing predictions through an aggregation strategy (see below) that conveys both consistency and uncertainty across taxa.

**Figure 1.**
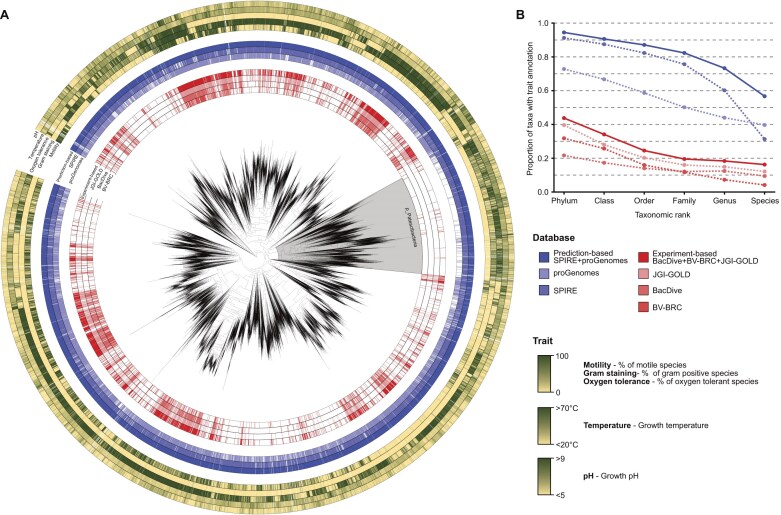
Experimental and predicted trait coverage across the bacterial tree of life. (**A**) Phylogenetic tree of 23 112 bacterial genera based on GTDB taxonomy release r220. From the innermost ring outward, red strips indicate genera for which experimentally derived trait information is available from databases such as JGI GOLD, Bac*Dive*, and BV-BRC, while blue strips indicate genera with traits predicted from genome sequences using proGenomes and SPIRE. Subsequent concentric gradient rings represent the proportion of species in each genus exhibiting the following traits: motility, Gram staining, oxygen tolerance, growth temperature, and growth pH. The gray shade highlights the phylum Patescibacteria, for which experimentally derived trait data are available for only 17 of 4581 species (0.37%), whereas prediction-based approaches provide trait annotations for 1677 species (36.7%). For genus-level trees, the lowest common ancestor (LCA) of all species within each genus from the GTDB-species tree was designated as the genus node, which was treated as a leaf in the reconstructed tree. As the GTDB taxonomy was used, all the LCAs were monophyletic with respect to their corresponding genera by definition. The resulting trees were visualized using iTOL [26]. (**B**) Proportion of GTDB taxa with trait annotations across taxonomic ranks. Blue lines represent coverage from prediction-based sources (proGenomes, SPIRE, and their combination), and red lines represent coverage from experimental sources (Bac*Dive*, BV-BRC, JGI GOLD, and their combination). Prediction-based methods consistently achieve broader coverage than experiment-based sources across all ranks.

Genome-based trait predictions were generated for 906 855 high-quality (CheckM2 [22] completeness > 90%, contamination < 5%, and GUNC [23] passed) isolate genomes from proGenomes3 [24], and 1 102 679 medium- and high-quality (CheckM2 [22] completeness > 50%, contamination < 5%, and GUNC [23] passed) MAGs from SPIRE [25]. In total, this approach yielded 207 million trait predictions. By incorporating MAGs derived from environmental samples, the dataset encompassed a broader phylogenetic diversity, enabling trait coverage across a wider range of taxa (Fig. 1A and B). Notably, the increase in trait annotation coverage relative to experiment-based datasets was particularly pronounced in clades that are rarely isolated or cultivated, such as Patescibacteria and Nanoarchaeota (Fig. 1Aand Supplementary Figure S1).

Overall, metaTraits captures more than 144 traits (2855 if individual chemical compounds are counted separately) that are organized into 46 groups and observed across 64 126 named species in GTDB r220 (104 722 by SPIRE clusters, 54 654 by NCBI taxonomy), representing the largest publicly available collection of microbial phenotypic trait data to date.

### Data standardization

To enhance interpretability and machine-readability, trait names were mapped wherever possible to standardized vocabulary terms. The Ontology of Microbial Phenotypes (OMP, [27]) served as the primary framework for capturing microbial phenotypic traits and experimental observations, supplemented as needed by terms (including composites thereof) such as from MICRO [28], SNOMED [29], and Gene Ontology (GO, [30, 31]). For each mapped term, metaTraits provides a direct link-out to the corresponding Ontology Lookup Service (OLS, [32]) page, enabling users to easily explore definitions and relationships for each trait.

For taxonomy harmonization, we used taxonkit [33] via pytaxonkit [34] to assign standardized taxonomy identifiers for both NCBI ([35], 2025-07-28) and GTDB ([36], release r220). As there is no official taxonomic identifier system for GTDB, we employed the GTDB taxdump (gtdb-taxdump v0.5.0 r80-r220) provided by taxonkit for consistent mapping across the dataset. Not all trait records were annotated with both NCBI and GTDB taxonomy, or had an associated genome for taxonomic classification. However, since we had a large set of fully classified genomes, we created a mapping between the two systems: for each taxon in one taxonomy, a corresponding taxon in the other was assigned if at least 85% of genomes shared the same ID in both. This approach, with an average agreement rate of 99.47% (GTDB to NCBI) and 99.87% (NCBI to GTDB), respectively, enables robust cross-referencing between NCBI and GTDB taxonomies, which can be useful for many applications beyond trait analysis.

## Database content

### Website

The metaTraits website (https://metatraits.embl.de) centralizes trait data in a unified and accessible resource. Users can search and explore the database using either NCBI or GTDB taxonomy, with the option to flexibly include or exclude specific data sources depending on the specific research requirements (e.g. to focus on culture-derived or genome-based predictions). Trait data are aggregated by taxonomy, enabling trait summaries from species to phylum level and displaying distributions within relevant context. To reflect underlying genomic variability, numerical traits are summarized by their median, while binary and categorical traits report the fraction of observations assigned to each class, along with the total number of contributing observations and databases. When trait data for a clade are consistent (≥85% of observations in one category), a summary trait label is provided. This aggregation strategy also makes prediction uncertainties visible: inconsistent predictions within a clade result in broader distributions, and when fewer than 85% of genomes agree on a state, the trait is flagged as “no robust majority.” Each trait estimate is linked to its original evidence in the source databases, ensuring transparency and verifiability, and additional link-outs to relevant external resources are available. Downloadable taxonomy-level summaries support broad accessibility and seamless integration into downstream analyses.

### Annotation of user-submitted data

Two annotation workflows for user-submitted data extend the practical utility of metaTraits:

Genome annotation: The Nextflow-based porTraits workflow predicts microbial phenotypic traits for user-submitted isolate genomes and MAGs by integrating multiple genome-based prediction tools. Users provide genome or MAG FASTA files as input, porTraits calls genes with Prodigal [37], generates KO and PFAM matrices via eggNOG-mapper [38], and computes trait predictions using the models from Bac*Dive*-AI, Traitar, and MICROPHERRET; GenomeSPOT is run directly on the genome input. Taxonomic assignments are obtained using reCOGnise (for NCBI taxonomy) and GTDB-Tk [39] (for GTDB r220), which also enable retrieval of similar trait records from the metaTraits database for contextualization. For NCBI taxonomy assignment, reCOGnise extracts mOTUs [40] markers with fetchMGs [41], aligns them to the COG database using MAPseq [42], and assigns taxonomic IDs. The porTraits workflow can be executed directly on the metaTraits website (no registration required), via the interface of the Cloud-based Workflow Manager (https://clowm.bi.denbi.de, [43]), or their API. All workflow code is openly available at https://github.com/grp-bork/porTraits.
Microbial community annotation: This workflow enables users to annotate entire taxonomic profiles of microbial communities with trait information. It parses outputs from commonly used taxonomic profiling tools, maps features to taxonomic IDs, and annotates taxa with trait data from selected sources. Supported profilers currently include mOTUs [44], MetaPhlAn [45], Kraken [46], Krakenuniq [47], Bracken [48], Kaiju [49], as well as generic OTU tables with matching taxonomies.

These workflows make metaTraits not only a comprehensive reference resource but also an integrative tool compatible with widely adopted microbiome analysis software.

## Use cases and outlook

**Figure 2.**
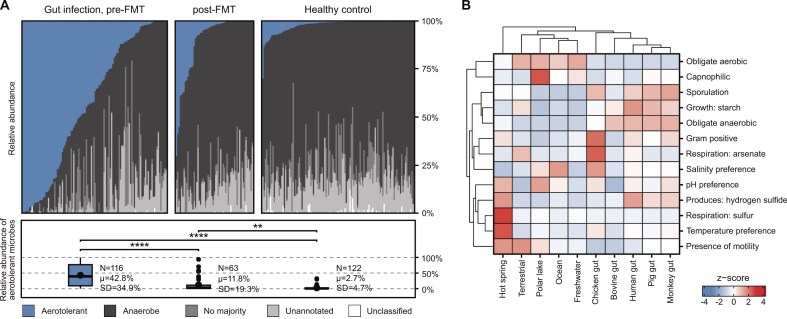
Trait-based comparisons of microbial communities using metaTraits. (**A**) Compositional shifts in microbes with distinct oxygen preference in the human gut (data from Metalog [51]) among individuals with *Clostridioides difficile* infection before and after fecal microbiota transplantation (FMT), compared to healthy controls. Bars show community-level relative abundances of aerotolerant (blue) and anaerobic (dark gray) taxa, as well as those that are taxonomically unclassified, lack trait annotations, or have no annotation majority. The data reveal a marked enrichment of aerotolerant and depletion of obligate anaerobic microbes during infection, which reverses following FMT. (**B**) Trait distributions across microbiomes from aquatic and terrestrial environments, and various animal hosts (data from Kim *et al.* [4]). Shown are *z*-scored trait abundances across habitats for selected traits, including Gram staining, sporulation, motility, and preferences for oxygen, temperature, and pH. Data are summarized across samples within each habitat, reflecting distinct ecological and physiological characteristics of the microbes and their environments.

metaTraits provides a foundation for diverse research applications and integration into existing microbiome analysis workflows. Key use cases include:

- **Quick taxonomic trait characterization:** Researchers can rapidly look up and summarize microbial traits at multiple taxonomic levels. This is especially valuable for studies based on 16S rRNA gene amplicon data, which often have resolution limited to the genus level. The ability to query both NCBI and GTDB taxonomies within metaTraits further extends utility to a broad user base.
- **Distribution and evolution of traits:** Microbiologists can assess whether traits observed or predicted for their strains are typical or unusual within their phylogenetic context, and readily identify exceptions or outliers within lineages. Overlaying traits with phylogenetic trees can provide insights into their evolution (e.g. motility as shown in Fig. 1A).
- **Community trait annotation:** Microbiome researchers can annotate entire microbial communities with trait prevalence and abundance profiles, enabling analyses of functional and ecological patterns at the community level. As shown in Fig. 2, such comparisons reveal distinct trait distributions across ocean, hot spring, soil, and host-associated microbiomes (Fig. 2B) and highlight functional shifts in health contexts, such as the increased prevalence of oxygen-tolerant microbes during infection (Fig. 2A). These profiles therefore facilitate the study of associations between microbial functions, taxa, and environmental variables.
- **Cross-database integration:** Integration with resources like SPIRE [25], GMGC [50], and Metalog (https://metalog.embl.de/) supports comprehensive multi-omic analyses, enabling researchers to relate traits and trait profiles to community composition, gene prevalence, and host or environmental data across databases.
- **Traits in context:** By combining traits of genomes or microbial communities with rich contextual metadata from resources like Metalog, researchers can investigate how functional traits are distributed across ecosystems, biogeography, disease, or health status. For example, one could explore whether certain traits are more common in gut microbiomes of individuals with inflammatory bowel disease, or how metabolic strategies differ between marine and freshwater samples. Ecologists can also utilize trait information to build and test hypotheses about the ecology and biogeography of prokaryotes.

Future updates to metaTraits will expand trait coverage, incorporate new prediction tools and updated genome catalogs, and enhance interoperability with external databases, supporting the continued growth of trait-based microbiome research.

## Supplementary Material

gkaf1241_Supplemental_File

## Data Availability

All raw data underlying metaTraits v1 is publicly available via proGenomes (https://progenomes.embl.de/), SPIRE (https://spire.embl.de/), and the culture databases listed in Table 1. No new sequencing data were generated for this study. The derived and curated data described above are freely accessible and downloadable via https://metatraits.embl.de. No registration is required. metaTraits is released under a Creative Commons Attribution-ShareAlike 4.0 International License. Source code for the trait annotation workflow porTraits is available at https://github.com/grp-bork/porTraits and on Zenodo (10.5281/zenodo.16809307).

## Notes

Present address: Koonkie, Vancouver, Canada
