# Supplementary material for: metaTraits: a large-scale integration of microbial phenotypic trait information
Source: Nucleic Acids Res. 2025 Nov 26;54(D1):D835–41. doi: 10.1093/nar/gkaf1241 (PMC12807735; doi:10.1093/nar/gkaf1241)
Supplement: gkaf1241_Supplemental_File [file gkaf1241_supplemental_file.pdf]

Supplementary Figure

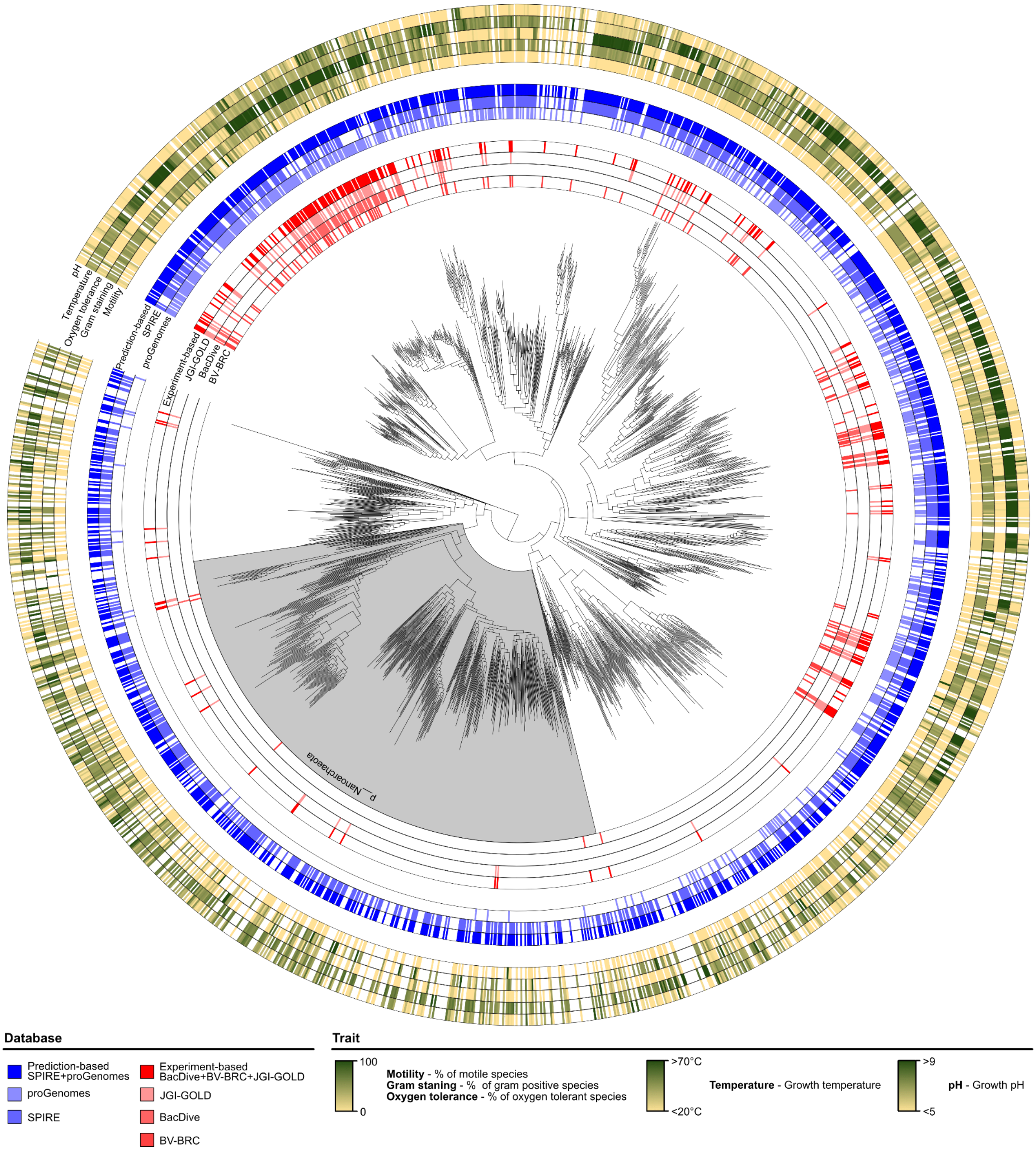

Supplementary Figure 1 | Phylogenetic tree of 1,847 archaeal genera based on GTDB taxonomy release r220. From the innermost ring outward, red strips indicate genera for which experimentally derived trait information is available from databases such as JGI GOLD, Bac*Dive*, and BV-BRC, while blue strips indicate genera with traits predicted from genome sequences using proGenomes and SPIRE. Subsequent concentric gradient rings represent the proportion of species in each genus exhibiting the following traits: motility, Gram staining, oxygen tolerance, growth temperature, and growth pH. The grey shade highlights the phylum Nanoarchaeota, for which experimentally derived trait data are available for only 9 of 847 species (1.06%), whereas prediction-based approaches provide trait annotations for 235 species (27.7%).
